# Augmented Reality Games and Presence: A Systematic Review

**DOI:** 10.3390/jimaging8040091

**Published:** 2022-03-29

**Authors:** Anabela Marto, Alexandrino Gonçalves

**Affiliations:** Computer Science and Communication Research Centre (CIIC), School of Technology and Management (ESTG), Polytechnic of Leiria, 2411-901 Leiria, Portugal; alex@ipleiria.pt

**Keywords:** augmented reality games, immersion, presence

## Abstract

The sense of presence in augmented reality (AR) has been studied by multiple researchers through diverse applications and strategies. In addition to the valuable information provided to the scientific community, new questions keep being raised. These approaches vary from following the standards from virtual reality to ascertaining the presence of users’ experiences and new proposals for evaluating presence that specifically target AR environments. It is undeniable that the idea of evaluating presence across AR may be overwhelming due to the different scenarios that may be possible, whether this regards technological devices—from immersive AR headsets to the small screens of smartphones—or the amount of virtual information that is being added to the real scenario. Taking into account the recent literature that has addressed the sense of presence in AR as a true challenge given the diversity of ways that AR can be experienced, this study proposes a specific scope to address presence and other related forms of dimensions such as immersion, engagement, embodiment, or telepresence, when AR is used in games. This systematic review was conducted following the PRISMA methodology, carefully analysing all studies that reported visual games that include AR activities and somehow included presence data—or related dimensions that may be referred to as immersion-related feelings, analysis or results. This study clarifies what dimensions of presence are being considered and evaluated in AR games, how presence-related variables have been evaluated, and what the major research findings are. For a better understanding of these approaches, this study takes note of what devices are being used for the AR experience when immersion-related feelings are one of the behaviours that are considered in their evaluations, and discusses to what extent these feelings in AR games affect the player’s other behaviours.

## 1. Introduction

Among the latest computing technologies disseminated across society, we find virtual reality (VR), augmented reality (AR), and the concept of mixed reality—the avant-garde of interactive experiences. Thus, for developers and researchers in the area of all these extended realities, these are frequently drawn on to improve various applications in several fields.

According to our research in recent years, with AR implementations and experiences, in addition to their irrefutable advantages, a vast number of questions have arisen when trying to provide objective data related to these types of applications.

A previous study conducted a literature review focused on VR and AR systems and their effects on learning [1]. This state of the art consisted of several categories including VR and AR games, mainly serious games and simulation games. According to the literature review they conducted, AR games are found to have positive effects on students’ motivation and attention, and on students’ learning and critical thinking. They focused on the technology used and not on the type of system developed. Notwithstanding its value, we consider that more insights are needed for a fruitful use of AR. Given the importance of content in AR, as well as the design and interactivity of AR systems in the users’ sense of presence, the current study aimed to collect and summarise the results obtained from AR games. The various advantages found in this for AR and VR applications are so vast and varied that a large number of new questions have been raised. This literature review aims to provide particular answers and is specific enough to focus on one technology—AR—and on one type of application—games. Considering these two topics, we searched for all AR games and carefully analysed not only what the role of presence has been in these implementations but also those of immersion, engagement, embodiment, or co-presence. In sum, immersion-related feelings were contemplated.

The current systematic review provides a detailed analysis of immersion-related feelings in AR games and aims to answer the questions below. Note that, in order to identify feelings that may be related to immersion, the term “immersion-related feelings” is considered in the current study to designate the most common states that may be related to immersion, such as presence, engagement, embodiment, or telepresence.

This systematic review aimed to answer the following questions:

- Q1: What are the immersion-related feelings that are being reported in AR games?
- Q2: How are immersion-related feelings being evaluated in AR games?
- Q3: What are the devices that are being used for AR games when immersion-related feelings are being considered in the experience?
- Q4: To what extent do immersion-related feelings in AR games affect other feelings and behaviours in the player?

The following subsections provide the context and clarify why this review is needed for all those who intend to use and explore augmented reality solutions.

### 1.1. An Overview of the Technology

An overview of augmented technology is presented as well as an outline of AR game considerations.

#### 1.1.1. Overview of Augmented Reality Technology

AR has undergone rapid growth in terms of technological advances and gaining popularity across the world, becoming a frequent approach in different areas for different purposes. From medical aid to learning experiences at schools and at museums to pure entertainment, AR is an interesting and attractive way to innovate in the digital world. In this case, since AR can be considered a variant of VR and as Ronald T. Azuma sustained back in the 1990s [2], AR can be used to blend innovation in the digital world with real surroundings. Several definitions of AR have been disseminated, including some variations of the technology as being mixed reality or extended reality. This study does not intend to disclose the various definitions nor to compare the differences between them, whereas the definition of Azuma, widely adopted across the literature, stands for the current study. Therefore, when AR technology is referred to, it is implicit that it refers to any system that combines real and virtual content regardless of the technology used as being an interaction in real-time in the three-dimensional world [2].

The technological development of the last decade brought new possibilities for AR to succeed as in the case of smartphones or head-mounted displays such as HoloLens. Studies aiming to enhance our understanding of the impact of AR largely support its usage as can be observed in systematic reviews focused on the advantages of AR in education [3], in medical training [4], in patients with autism [5], in industrial maintenance [6], in cultural heritage [7], etc. More generic studies related to the use of AR have also been found, such as the systematic review by Dey et al. [8] or the survey by Billinghurst et al. [9]. When analysing previous AR studies and applications, one frequently realises that those applications are games or gamified applications of AR. This raises new questions related to the type of content and interactivity that is implemented, especially when covering AR solutions, since this technology can also vary in the amount of virtual elements that are presented to the user—notably the virtuality continuum concept presented by Milgram et al. [10].

#### 1.1.2. Augmented Reality Games

Depending on the intended use of the AR implementation, it is important to understand how users react and what the impact of their experience with AR is. Some authors have highlighted the blurred boundaries in AR experiences between the activity itself and the surroundings as non-players and elements of the real space can affect the experience [11]. This challenge, however, was verified as an advantage in a previous study that stated that AR blurs the boundaries between virtual and real objects, resulting in a better sense of immersion [12]. To date, it is undeniable that using AR can be beneficial, but it is not clear how. Given the frequency of creating game scenarios for engaging experiences when using AR and the lack of knowledge surrounding how games and AR are succeeding among the scientific community, this study proposes a systematic review of AR games. The most referred AR game to date is Pokémon Go, on which several studies have carried out interesting research [13,14,15,16]. Nonetheless, AR games have become a matter of interest for the scientific community in the last decade. The reasons for which games are frequently implemented vary, but according to the literature, there are reasons for this. The positive effect of learning goals in games is validated in the literature as the systematic review shows [17]. In addition to that, it has been demonstrated that varied aspects of engagement and satisfaction are widely related to games, as can be observed in a systematic review focused on engagement in digital entertainment games [18].

A systematic review targeting serious games has been partially undertaken, yielding useful insights for all those who intend to develop a game for learning purposes in science education [19]. The reduced scope of this study suggests that other genres of games should also be analysed and compiled to help further implementations in becoming more accurate and objective. AR technology used for gaming with mobile devices is said to become a new gaming experience for players while enhancing their immersion [20]. Moreover, mobile AR has been highlighted as an essential immersive innovation [21]. Note that it has been stated in the literature that a way to increase user engagement with the application is to use AR [22]. Furthermore, as we review previous studies, we start to create a connection between AR, games, and immersion. Thus, to create an AR game that provides a good immersive experience for the user, it is not clear how to conduct and implement such a game. In contrast to aforementioned literature reviews, e.g., for serious games [19] or for engagement in digital entertainment games [18], none have been found for AR games. In order to make this literature review objective, one goal of this research study was to provide insights related to the devices that are being used for AR games when immersion-related feelings are a variable considered in the experience (Q3).

### 1.2. Immersion-Related Feelings

This subsection aims to provide context on immersion feelings, including feelings that may be related to immersion, such as the sense of presence, engagement, embodiment, or engagement with the experience. Initially, an overview of the different definitions of immersion is presented, with the aim to provide some context of the different feelings that may be related to immersion. Then, some of the determinants that may affect immersion are clarified and finally, evaluation insights are exposed.

#### 1.2.1. Overview of Immersion-Related Feelings

According to the literature, immersion is a powerful experience of gaming and it is frequently regarded as a significant matter of interaction [23]. Jennett et al. [24] referred to immersion as being related to the specific and psychological experience of engaging with a computer game as clearly distinct from the common definition of engaging experiences that considers the flow, the cognitive absorption, and the presence. In their detailed study on immersion in games, it has been demonstrated that the outcome of immersion may be independent from the outcome of the game itself and yet, immersion is the result of a good gaming experience. According to Laurie Taylor [25], immersion in computer games can be related to immersion in activities of the game, or related to immersion as an illusion of being in the game environment. The second type of immersion is known as the sense of presence. As we probe deeper into the definition of immersion, it is noticeable that this concept can bear a variety of meanings, mainly depending on the authors and on the activities that are being performed. Immersion is commonly linked with the concepts of presence, suspension of disbelief, or transportation, and are terms that share certain characteristics that are in line with the expectations of contemporary media [26].

Anyhow, the scope of this research is not to navigate across the different definitions of immersion, given that this has already been established in previous studies [24,27]. Rather, this study focused on AR games that consider immersion-related feelings in their studied variables and intended to cover variables and/or constructs that are being used to perceive immersion or presence in the experience. Thus, the sense of presence is also considered for the current systematic review, as well as embodiment, engagement, social presence and telepresence. The decision to include engagement—which may take a wider inclusion of feelings that may not be related to immersion—is due to its relation to game involvement in the Game Engagement Questionnaire [28]. In line with this approach, for the current study’s analysis, when engagement is considered as a variable under study, it is related to the mode of being present in connection with the surroundings, as in [29].

When observing presence studies, frequently considered for immersion-related studies, it is important to mention that the definition of presence is still under substantial discussion and, as explained above, it is not the purpose of this study to discuss the different definitions provided in the literature. This section aims to provide some context to better understand the analysed studies in the future. The sense of presence can be related to the sense of being in a virtual element [30]. However, it has also been stated that presence in a virtual environment does not require a displacement of attention from the physical place [31]. These two approaches are relevant in the context of AR since the addition of virtual elements into a real environment may vary depending on each implementation—again, the virtuality continuum concept presented by Milgram et al. [10] helps understand the virtuality and reality of an AR experience. It has been supported that blurring the line between virtuality and reality in AR games can result in an enhanced sense of presence [27].

In line with this insight, and as highlighted in the literature, to explore how different realities may be blended and how people feel present is foundational [11]. Furthermore, knowing that the differences between real and virtual experiences are filtered by users’ actions rather than the environment itself [32], and that presence is also affected by social and cultural dimensions [33], it is necessary to include social presence and co-presence, and link them to these feelings of presence and immersion as they are directly related to the AR game experience itself. Note that several applications demonstrated the benefits of adding virtual humans (or avatars) [34] and social presence and co-presence are constructs frequently used to measure users’ perception of virtual humans in these types of implementations [35].

This brief overview of immersion-related concepts demonstrates several directions that immersion-related feelings may take. Participants often confuse the concepts of presence with immersion in their responses when participating in this type of study [36]. Thus, this study will provide an answer for what immersion-related feelings are being reported in AR games (Q1).

#### 1.2.2. Determinants of Immersion-Related Feelings

Immersion-related feelings may vary according to a wide variety of factors presented in the literature such as realism [31], interactivity with the virtual system [37], and the design of the game in first person mode or stereo sounds [38]. Moreover, the size of the viewpoint, the control of a player over viewpoints, and the quality of the graphics [37] have been linked to immersion-related feelings. Audio is also considered an important aspect of the experience—not only as having the characters talk directly to the player but also as an environmental narrative [39]. In addition to these factors, the semantic coupling between the physical space and the virtual narrative may affect experienced immersion by integrating real cues related to the virtual narrative [40]. In line with the factors presented, feelings of immersion in AR frequently encompass categories such as interaction, interference (as being the interaction between the physical environment and the AR environment), tactile experience, or moving in the environment [41]. Thus, the current study aimed to understand the extent to which immersion-related feelings in AR games affect other feelings and behaviours in the player (Q4).

#### 1.2.3. Evaluating Immersion-Related Feelings

According to Rebollo et al., immersion in AR games triggers the feeling of being physically located in the place where players are playing the game [22]. Immersion-related feelings are known to positively affect several behaviours or outcomes in AR experiences, such as improving learning environments [42], increasing engagement [22] and enjoyment [13], or are considered an essential factor for positive game experiences [43].

This study is important to clarify the relationship between AR and immersion. Across the literature, it is common to conduct evaluations related to immersion using similar procedures and tools that are being used for VR environments. However, as indicated in the literature, studies of AR immersion-related feelings have revealed contradictory results when evaluated and compared to VR approaches. For example, while participants pointing to something can be interpreted as an indicator of presence, according to Slater et al. [44] within their VR studies, it has contrastingly been demonstrated to strongly differ in an AR presence study where “pointing to virtual content” was negatively correlated to the sense of presence [45].

The lack of validated, theory-based instruments for measuring immersion is well known in the literature, and as Georgiou et al. highlighted, it is even more evident for AR implementations [46]. These authors conducted a study and proposed an instrument for measuring immersion in location-based AR settings: the augmented reality immersion (ARI) questionnaire. They carefully analysed precious literature strategies for evaluating immersion and AR approaches as well as underscored that game-based studies seeking to operationalise and measure immersion are still inconclusive, which has given rise to new questions and issues about AR games and immersion. For this reason, the current study aimed to answer the question of how immersion and related feelings are being evaluated in AR games (Q2).

## 2. Methods

The current literature review followed the PRISMA method [47] and then considered the updated version PRISMA 2020 statement [48] as a guide to the development of systematic reviews so as to ascertain a transparent and complete reporting of the surveyed topics.

### 2.1. Eligibility Criteria

A randomised search of AR games that considered immersion-related feelings in their studies was performed, with the aim to provide relevant data taking into account its implementations, usage, and evaluation. The inclusion criteria are specified in Table 1, whereas the exclusion criteria are outlined in Table 2.

### 2.2. Examination Strategy

The strategy followed to obtain a full analysis regarding the specified topics included two search stages: a search of online searches, and a search analysing the records obtained from the previous search stage.

For the first search stage, the literature was identified through online searches by conducting an extensive search with the EndNote 20 software, through the Science Citation Index on Web of Science (Clarivate) conducted on 3 February 2022. The search was performed to be equivalent to the following logical expression: Title/Keywords/Abstract containing (“Augmented Reality” OR “AR” OR “Mixed Reality” OR “extended Reality” OR “XR”) AND (“Game” OR “Games”) AND (“Immersion” OR “Engagement” OR “Presence” OR “Embodiment” OR “Telepresence”).

**Figure 1:**
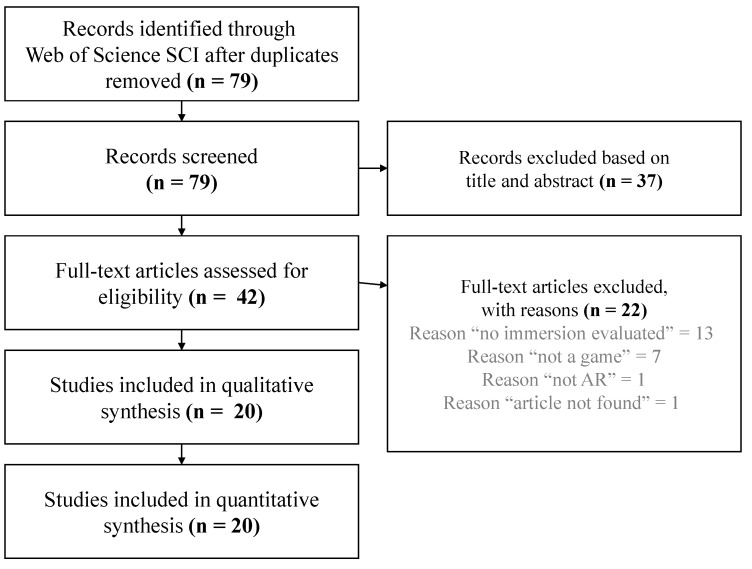
Summary of the identification, screening, eligibility, and inclusion of studies according to including and exclusion criteria.

For the second search stage, carried out when reviewing full-text articles (see Figure 1 for a better context concerning the several steps proposed in PRISMA method), a search was performed for eligibility when analysing the full-text articles, since some articles did not provide enough information for qualitative or quantitative analysis. In addition, the Google Scholar platform was used to make an oriented search for studies that seemed to meet the established inclusion criteria when analysing the state of the art of full-text articles obtained in the referred search.

### 2.3. Study Selection

Following the aforementioned search strategy, whereby a total amount of 79 records were obtained, an eligibility assessment was performed independently in a conventional unblinded standardised manner. Each paper was reviewed by two reviewers to decide its eligibility based on the title and abstract of each study taking into consideration the exclusion criteria. When a record was rejected by one reviewer and accepted by the other, that record was kept for eligibility.

When performing this task, 37 records were discarded, and the remaining 42 full text papers were assessed for eligibility.

#### Data Collection Process

The eligibility assessment performed across 42 full-text papers aimed to collect all the data needed for the current systematic review. During this process, some articles were dropped—considering the exclusion criteria—and none were added—after performing the second search stage.

A total of 22 articles were excluded from the analysis when screening the text due to not presenting immersion data (13 records), not being a game (7 records), not presenting an AR experience (1 record) or for not being possible to obtain the full-text version of the record (1 record). After this process, a total of 20 studies remained for the qualitative and quantitative analyses.

## 3. Results

After removing all duplicated records, the search performed in the identified database returned 79 articles. The titles and abstracts of the unique 79 records were analysed, taking into account the eligibility criteria, among which 37 records were excluded for not meeting such criteria. As a result, 42 records were obtained as eligible for a full-text analysis. Among those 42 records, 22 more records were excluded based on the previously defined exclusion criteria, resulting in a total of 20 studies carefully analysed for this survey. Please refer to Figure 1 for a detailed overview of the study selection.

### 3.1. Qualitative Analysis

This first subsection of results depicts the most relevant qualitative data gathered. A list of the analysed studies is presented, a word cloud based on the titles was generated, and an overview of the major findings is emphasised in this subsection.

The analysed articles included in this systematic review are listed in Table 3. The search returned results from after the year 2011 and an increasing number of studies per year were identified—from the 20 records starting in 2011, more than half were found after the year 2020, i.e., within the last two years.

Several other studies seemed to fit the inclusion criteria as they presented interesting approaches in AR games such as a game targeting aesthetic engagement with a city [29], an AR adventure game focused on communicating the history of a heritage location to casual visitors [39], or a survey that examined the impact of COVID-19 related to social restrictions on the physical and mental well-being of AR game player [61]. Despite the interest and positive insights shared by these authors among several others, they were not included in the systematic analysis as none of their results were related to immersion nor presence.

On the other hand, some interesting studies investigated immersion-related feelings in their work, such as the supernumerary hand illusion [62], the informal science learning study focused on engagement factors commonly associated with the use of AR [63] or a research focused on raising the level of engagement as a pedagogical advantage [1]; however, they did not present or use an AR game, and were consequently excluded from the current systematic review analysis.

#### Word Cloud

**Figure 2:**
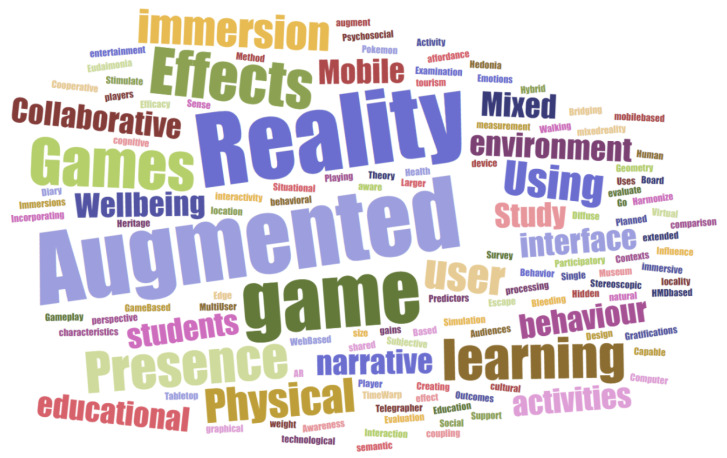
Word cloud generated from the titles of the 20 records analysed for the current study with the aid of the online word cloud generator Jason Davies.

Considering the 20 records which were carefully analysed, a word cloud was generated based on the titles of those studies, which is presented in Figure 2. This word cloud was generated with the aid of the online platform Jason Davies. More info about the word cloud generator Jason Davis may be found at the following webpage: www.jasondavies.com (accessed on 28 Febrary 2022).

Regarding the technical concepts and approaches used, we observed a preference for “augmented reality” while “Games” and “game” were predictably frequent words as well. There was a tendency for the frequent use of “presence” and “immersion”. An interesting concept that appeared quite frequently was “learning”.

### 3.2. Major Findings and Conclusions

The major findings are highlighted hereinafter.

#### 3.2.1. Collaboration AR Games

McCall et al. [11] reported that the presence feelings of a co-player approach were unsurprisingly higher than the non-collaborative experience. Clear links to reality in the experience were reported as being slightly more critical for presence feelings, while audio elements were reported to contribute to a stronger sense of presence than the graphical elements. They also sustain that the nature of tasks and actions define players’ sense of presence.

Following the study by McCall et al. [11], Putten et al. [45] proposed to clarify which behavioural elements could correlate with the player’s presence. Contrary to what previous literature related to VR has stated, they claimed that in their AR experiment, pointing behaviour and verbal feedback towards virtual elements are negatively correlated to the sense of presence perceived by the user. Their results showed that collaborative gameplay had no influence on presence but virtual interaction and social presence, however, evinced a strong sense of presence.

Lee et al. conducted their study in an identical collaborative scenario to understand the effects of latency [35]. Their results showed a strong negative effect of latency on the perceived co-presence. Nonetheless, although the overall patterns are similar, they noticed that the sense of co-presence is less affected by latency than by causality or realism.

#### 3.2.2. Varying Devices and Scenarios

Datcu et al. also used a collaborative game to compare presence in physical and AR environments [41]. Their results showed that AR players reported lower levels of presence and situational awareness. The authors highlighted the lower interaction felt by AR participants concerning the interaction in the physical environment.

In a serious game approach, Furió et al. [50] studied the effects of the size and weight of mobile devices for children between 8 and 10 years old comparing the AR experience using two devices. Their results showed that tablet PCs obtained a marginally higher score in engagement than iPhone users, however, the identified difference did not influence the children’s engagement. Aiming to ascertain the interaction effect between individual cognitive styles and technological context, devices were also compared in the study by Raptis et al. in which immersion was a variable under study [52]. Their results showed that immersion is significantly higher with mixed reality approaches than when using a computer without AR experience. Immersion was also stated to be affected by cognitive style, i.e., individual differences.

Furthermore, in their study focused on devices, Sekhavat et al. compared the results obtained from different types of camera, namely VR, AR, and stereo-AR [27]. They observed that using the AR cameras and the stereoscopic AR camera can result in a better sense of immersion in players in comparison to the VR camera. No differences were found when comparing the experience with an AR camera with the stereo-AR camera.

#### 3.2.3. AR Games Activity and Social Presence

Koh et al. [13] collected results on playing the game Pokémon Go while walking. They identified immersion as a significant negative predictor of intention to play while walking. Walking while playing was also covered by Kosa et al. in their study on presence in AR games and how it is related to daily physical activity levels and the well-being of players [60]. They demonstrated that presence was associated with daily walking duration and daily subjective vitality, suggesting that presence could be an important factor for engagement in AR games. They also supported the importance of emotional and narrative presence, giving less importance to physical presence.

Social presence as being an item of continuance intention—hedonic gratification—was studied by Bueno et al. also using the AR game Pokémon Go as a case study [15]. In their study, they disclosed that social presence has a positive impact on the continuation of ones’ intention to play Pokémon Go. In an interesting approach relating to social behaviours, Krzywinska et al. also raised the question related to which methods may be necessary to effectively achieve social immersion in cultural heritage installations [26]. However, no related results have been provided to date since only a usability test was conducted.

Social presence was also the focus in the research study performed by Oriti et al. which compared interfaces. They observed that the AR interface obtained lower scores when compared to the VR interface, and noticed that behavioural involvement was higher for VR. Despite these insights, they realised that both interfaces obtained relatively low results for social presence in general [57].

#### 3.2.4. Story and Narratives in Immersion Feelings

Aiming to understand the role of AR to stimulate students, Estudante et al. observed that students are immersed in the story by being able to use AR games as a teaching method in a manner complementary to traditional methods [53]. Georgiou et al. also dwelt on the narrative as a variant condition to better understand its role in immersion [58]. Their findings showed increased immersion results for the participants submitted to a strong coupling between the narrative and physical space, when compared to the condition without relations between the AR activity narrative and the physical space. In this experiment, the sense of flow was stronger than the sense of presence for the second condition described here, while the sense of presence was higher than the sense of flow for the first—indicating strong coupling between narrative and physical space condition.

Similarly, with the narrative as a determinant of the experience in mind, Jin et al. conducted a comparison between a natural user interface with a graphical user interface using AR-based head-mounted displays [59]. For this comparison, the authors proposed two AR experiences, one being a role-playing game (RPG) and the other without this type of interaction. They concluded that natural user interfaces obtained a better sense of presence for users without the RPG experience, while users with the RPG experience had a better sense of presence and an increasing narrative engagement with the graphical user interface.

#### 3.2.5. AR Games Linked to Other Feelings

Lin et al. linked the immersion feelings to effective learning and stated that, according to their results, learners were more deeply immersed during the AR gameplay, achieving more effective learning [54]. Engagement was also linked to learning activities in the study of Rossano et al. [55]. They verified that pupils felt a sense of being “drawn in” by the AR application, showing their interest in the learning activity due to the engagement felt. With a focus on engagement—more specifically, self-engagement—Oh et al. [51] compared two AR scenarios: a game-based approach and a non-game scenario. Their results showed that self-engagement showed no statistically significant difference between the two approaches.

Engagement—more specifically, game engagement—was an element studied for psychosocial well-being by Seaborn et al. [56]. Their results showed that game engagement was positively correlated with eudaimonic orientation—the meaningful experiences, personal growth, expressiveness, and self- actualisation—but no correlation was found between game engagement and hedonic orientation—related to positive affect, the desire for comfort and relaxation, and the pursuit of pleasure.

It was previously stated that a study linked usability with immersion without providing results [26]. Shin et al. linked usability to immersion and presence as well and demonstrated it [36]. They observed that when players feel a certain level of immersion and presence, they consider the AR game usable and participants feel emotionally engaged with it. They denoted that when empathy and embodied cognition are shifted by these feelings, it starts to influence the affordance arousing as well.

### 3.3. Quantitative Analysis

A quantitative analysis is now presented, highlighting devices used in the AR games studies analysed herein, as well as the variables that were under study and under what condition they were evaluated.

#### 3.3.1. Devices Used and Variables Studied for Immersion-Related Feelings

Table 4 identifies the devices used for each study analysed. The variables under study that were related to immersion feelings are also specified as independent variables (IVs) and dependent variables (DVs).

**Figure 3:**
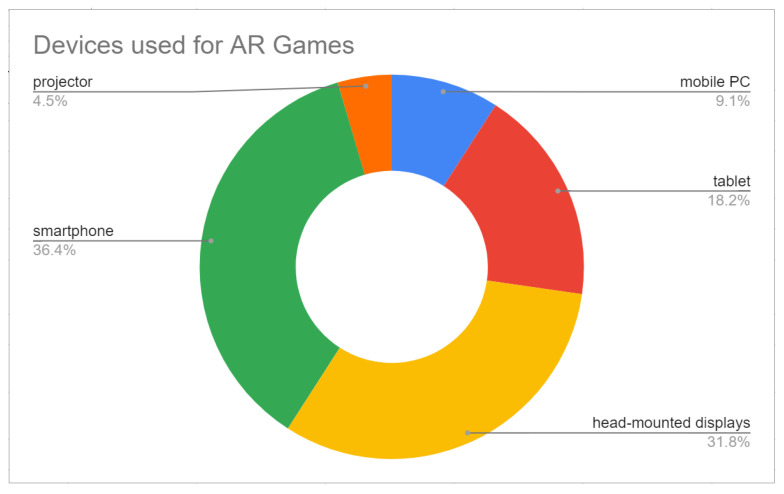
Summary of devices used for the AR studies analysed.

As illustrated in Figure 3, most of the studies used smartphones (36%) or head-mounted displays (32%). The other devices used were tablets (18%), mobile computers (9%), and a video projector (5%). Considering the platform of usage, smartphones and tablets may be grouped to represent 54% of the devices used. Since some studies implemented more than one solution, the percentages presented are related to a total of 22 implementations (all coming from the total of 20 studies analysed).

#### 3.3.2. Conducted Evaluations

In Table 5, the evaluation scenarios are summarised. The tools used for evaluation are presented, as well as the sample size from where the results were collected. When available, a reference to the used tool was also provided.

**Figure 4:**
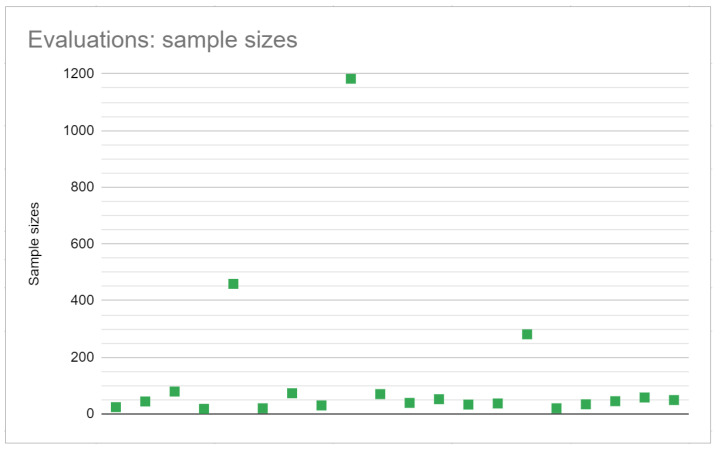
Sample sizes of the conducted evaluations for immersion-related feelings.

Following the data presented in Table 5, in Figure 4, the sample sizes of the conducted evaluations are illustrated.

## 4. Discussion

The discussion of this systematic review focuses on the questions previously presented in the introduction section.

### 4.1. Immersion-Related Feelings Evaluations

This research concludes that 90% of the analysed studies evaluated immersive-related feelings with questionnaires, since it was not clear which evaluation was used for the remaining 10%. Interviews and observations were used 20% and 10% of the time, respectively. The sample sizes of the conducted evaluation varied between 18 [41] and 1183 participants [15].

Most studies selected their evaluation tools based on validated questionnaires. This provides useful information for further evaluations.

#### 4.1.1. Immersion

Immersion was evaluated by using: (1) the Immersive Experience Questionnaire (IEQ) [24] twice—see [52] and also [27] for more information; (2) A custom questionnaire [69]—see [36] for more information; (3) the Augmented Reality Immersion (ARI) Questionnaire [46]—see [58] for more information.

#### 4.1.2. Presence

Sense of presence was evaluated by using: (1) a modified MEC spatial presence questionnaire [64] by adding some questions from a social presence questionnaire [65]—see [11] for more information; (2) an adapted version of the AR presence questionnaire [66]—see [41] for more information; (3) a custom questionnaire [69]—see [36] for more information; (4) presence questionnaire [31]—see [59] for more information; (5) the adapted player experience of need satisfaction (PENS) [68]—see [60] for more information.

#### 4.1.3. Engagement

Engagement had several approaches: self-engagement was evaluated by: (1) using a custom questionnaire [51]; (2) the short form of the User Engagement Scale (UES) [67]—see [55] for more information; (3) the Game Engagement Questionnaire [28], later changed to the Player Experience of Need Satisfaction (PENS) [68]—see [56] for more information.

#### 4.1.4. Social Presence and Co-Presence

Social presence was evaluated by using a custom questionnaire [15] while co-presence was evaluated using the Game Experience Questionnaire [70]—see [35] for more information.

### 4.2. Determinants of Immersion-Related Feelings and Outcomes

It was observed that the sense of presence is positively affected by audio elements, the nature of tasks and the actions of the player in the game, while realism may negatively affect presence [11]. While collaborative gameplay appears to not influence the sense of perceived presence, interaction and social presence evinced a strong sense of presence [45]. When studying experience environments, it was revealed that presence was lower in AR environments when compared to a physical environment [41].

Engagement appears to not be influenced by the device used when comparing a tablet to a smartphone [50]. This is an interesting outcome because the size of the screen has been frequently pointed out for other types of applications—not games—as a relevant factor for a good experience [72].

Immersion has been reported as being higher in AR scenarios than in non-AR virtual scenarios [52]. In line with this conclusion, VR cameras also appeared to be less immersive when compared to AR cameras and AR stereo cameras, while no differences were found between the two types of AR cameras [27]. Immersion is a significant negative predictor of intention to play while walking [13], but presence appears to be associated with daily walking duration and is demonstrated as an important factor for engagement in AR games [60].

In general, social presence has a positive impact on the continuation of one’s intention to play Pokémon Go [15]; nonetheless, social presence obtained lower scores when compared to a VR interface [57].

### 4.3. Overall Discussion

As discussed in Section 4.1, the immersion-related feeling more frequently evaluated in the conducted analysis was the sense of presence. To answer the first question of this systematic review about what immersion-related feelings are being reported in AR games, in addition to presence, other feelings were also identified, such as social presence, co-presence, immersion, social immersion, engagement, and self-engagement.

According to analysed records, there are no guidelines available for tracing a methodology to evaluate immersion/related feelings in AR games. Different tools were used and a wide variety of sample sizes were identified. Questionnaires, however, seem to be undeniable tools for evaluation. Therefore, to answer the question “How are immersion-related feelings being evaluated in AR games?”, the answer is not standardised. Immersion-related feelings are evaluated in AR games with questionnaires—mainly validated questionnaires—some specifically targeting AR experiences, whilst others are being evaluated with tools targeted for VR or games.

When trying to understand what devices are being used for AR games when immersion-related feelings are being considered in the experience, i.e., question number three, it was observed that most studies opted for implementing them in mobile platforms. Smartphones and tablets together correspond to more than half of the evaluated games, followed by head-mounted displays with 32% of occurrences. Mobile computers were used in two situations and a video projector was used in one.

As discussed in Section 4.2, AR games evaluations that considered immersion-like sensations provided interesting outcomes. In line with the literature, the improvement of learning abilities can be achieved using AR [50,52,54], as well as boosting one’s intention to continue playing [13,51], or even providing better engagement to encourage the interest in the usage of the application [55]. Furthermore, the interaction interface may considerably affect the obtained results, as highlighted in some studies [27,35]. Immersion feelings in AR should be well planned to determine whether the immersion is too high and whether the action of the user requires some attention, which otherwise may negatively affect the usage of the game [13]. As observed, immersion-related feelings in AR games can affect a wide set of other feelings and behaviours in the player.

As for the limitations of this current systematic review, we addressed the main limitation which was the search conducted on a single database. According to the literature, in addition to the Web of Science and the underlying databases and the indices it contains, there were at least 13 other academic search systems that were identified as well suited for systematic reviews, such as the ACM Digital Library or Scopus [73]. Notwithstanding, the high criteria used on the Web of Science database, as was observed, reported high-quality studies which provided useful and valuable insights for the presented systematic review.

## 5. Conclusions

The current systematic literature provided a global overview of AR games that considered immersion-related feelings in their studies. The analysed data allow us to understand what immersion-related feelings are being reported in AR games and it is now clearer how immersion-related feelings are being evaluated in AR games. It was observed that a wide variety of users’ feelings related to immersion were also identified as links between these feelings with others, such as engagement or learning skills.

This systematic review also exposed the different approaches that were conducted to evaluate immersion-related feelings. The way it has been evaluated is similar across studies, frequently making use of questionnaires. Validated questionnaires or customised versions of validated questionnaires are commonly used, but several studies have opted to create their own versions of questionnaires which are not validated. Some of the validated questionnaires are being used to ascertain AR feelings even if previously validated for VR scenarios. The differences between immersion feelings and presence feelings are interesting and considerably different depending on the desired outcome. For example, when users felt more present while playing an AR game, they walked longer during the day [60], while immersion was identified as a significant negative predictor of intention to play while walking [13]. Following this analysis, we suggest a careful process for evaluating immersion-related feelings in order to clearly identify users’ feelings towards an experience. We strongly discourage the creation of customised questionnaires that have not been previously validated, as it has been found that participants often confuse immersion with presence [36] and it can provide misleading conclusions.

An identification of the devices used for AR games, when immersion-related feelings are being considered in the experience, was pointed out to support the use of different technologies, depending on the target of each game. For example, the size of the screen is often pointed out in the literature as a hampering factor to engage in AR experiences [72].

An overview of immersion-related feelings in AR games and how they affect other feelings and behaviours in the player was conducted, and it was observed that these feelings in AR games can affect a wide range of other feelings and behaviours in the player. Overall, these feelings have been linked to engagement, learning abilities, the intention to continue playing, and the interest in it.

Major findings were also outlined as the different outcomes observed in the analysed studies are interesting and useful for further implementations and research. The research herein also demonstrated that there are topics raising some new questions, especially related to immersion and presence variables, which reveal that there are several research opportunities for AR games in the scientific community.

## Figures and Tables

**Table 1:** Inclusion criteria.

Inclusion Criteria	Description
Inclusion criterion 1	The paper has the one of following terms in the title, abstract, or keywords: *“Augmented Reality” OR “AR” OR “Mixed Reality” OR “extended Reality” OR “XR”;* along with one of the terms: *“Game” OR “Games”;* and with one of the terms: *“Immersion” OR “Engagement” OR “Presence” OR “Embodiment” OR “Tele-presence”*.
Inclusion criterion 2	The paper provides an experience with AR.
Inclusion criterion 3	The paper provides a game experience.
Inclusion criterion 4	The paper includes an analysis related to immersion, presence, engagement, embodiment, or tele-presence.
Inclusion criterion 5	The paper is written in English, Portuguese, or Spanish.

**Table 2:** Exclusion criteria.

Exclusion Criteria	Description
Exclusion criterion 1	The paper does not provide an experience with AR.
Exclusion criterion 2	The paper does not provide a game experience.
Exclusion criterion 3	The paper does not consider immersion-related feelings insights.
Exclusion criterion 4	The paper is a technical report, an abstract, editor note, call for paper, or thesis.
Exclusion criterion 5	The paper is written in another language than English, Portuguese, or Spanish.
Exclusion criterion 6	The paper is not available.

**Table 3:** The summary of the analysed records referring to the main purpose of each study. Note that the game type column refers to the specifics of each game and not the genre since all of them are AR games according to the game genre categorisation of Scott Rogers [49].

Reference	AR Game	Game Type	Main Purpose
McCall, 2011 [11]	“TimeWarp”	A collaborative two-player puzzle game	Discusses the evaluation of an outdoor AR location game
Putten, 2021 [45]	“TimeWarp”	A collaborative two-player puzzle game	Examines whether and which behavioural elements correlate with the player’s sensation of presence
Furió, 2013 [50]	(Name not found)	A serious game	An educational game that combined AR minigames focused on children
Datcu, 2016 [41]	“The Tower”	A puzzle game	Studies the different perception of presence and situational awareness in a physical environment as well as an AR environment
Koh, 2017 [13]	“Pokémon Go”	A location-based game	Identifies the critical predictors of behavioural intention to play Pokémon Go
Oh, 2018 [51]	“ARfract”	A game-based simulation	Tests an AR simulation-based learning environment to be installed at a science museum with two approaches: one game-based and another non-game
Raptis, 2018 [52]	“HoloTour”	An item-collection game	Ascertains the interaction effect between individual cognitive styles and technological context in cultural tourism
Sekhavat, 2018 [27]	“Ladybug”		Studies the effect of augmented reality in increasing the sense of immersion in mobile games
Bueno, 2020 [15]	“Pokémon Go”	A location-based game	Studies social presence as being an item of continuance intention (hedonic gratification)
Estudante, 2020 [53]	“Discoveries of Ernest Solvay”	An escape game	Uses AR to stimulate students
Krzywinska, 2020 [26]	“Augmented Telegrapher”	An escape game	Studies mixed reality games for museum and heritage contexts
Lin, 2020 [54]	(Name not found)	AR-based board game	Studies the effects of incorporating AR into a board game for health education
Rossano, 2020 [55]	“Geo+”	A serious game	Uses AR to support geometry learning learning
Seaborn, 2020 [56]	(Name not found)	A cooperative puzzle game	Studies cooperative game for psychosocial well-being
Shin, 2021 [36]	“Batman Arkham”, “Pokémon Go”, “House of Dying Sun”, “Harry Potter”, “Wizards Unite”, “Zombie Run”		Investigates the impact of augmented reality on user affordance and the role of immersion
Oriti, 2021 [57]	“Harmonize”	A shooter game	Compares immersion games in VR and AR
Lee, 2021 [35]	(Name not found)	A table top game	Compares the gameplay between AR and VR
Georgiou, 2021 [58]	“Mysterious disease”	A serious game	Investigates the factors affecting immersion during a narrative-based AR intervention for environmental science learning
Jin, 2021 [59]	“AR Journey”		Compares the natural user interface and graphical user interface and their impact on users’ presence
Kosa, 2022 [60]	“Ingress”		Studies presence in augmented reality games and how it is related to daily physical activity levels and players’ well-being

**Table 4:** List of the analysed studies presenting the devices used for the AR studies and the variables that were studied.

Reference	AR Device	Immersion-Related Variables
McCall, 2011 [11]	Ultra-mobile PCs	IV: none DV: presence
Putten, 2012 [45]	Ultra-mobile PCs	IV: collaboration DV: presence
Furió, 2013 [50]	Smartphone; tablet	IV: device (tablet vs. iPhone) DV: engagement
Datcu, 2016 [41]	AR HMD (a modified headset)	IV: environment (AR vs. physical) DV: presence
Koh, 2017 [13]	Smartphone	IV: none DV: immersion
Oh, 2018 [51]	AR glasses	IV: game-based vs. non-game DV: self-engagement
Raptis, 2018 [52]	AR glasses (HoloLens)	IV: technological context (desktop vs. mixed-reality) DV: immersion
Sekhavat, 2018 [27]	Smartphone; Google cardboard for stereo-AR	IV: type of camera (VR vs. AR vs. stereo-AR) DV: immersion
Bueno, 2020 [15]	Smartphone	IV: none DV: social presence
Estudante, 2020 [53]	Smartphone	(not enough data)
Krzywinska, 2020 [26]	AR glasses (HoloLens)	IV: none DV: social immersion
Lin, 2020 [54]	Tablet	(not enough data)
Rossano, 2020 [55]	Smartphone	IV: none DV: engagement
Seaborn, 2020 [56]	Projector; tablet	IV: none DV: engagement
Shin, 2021 [36]	Smartphone	IV: affordances in the game DV: immersion; presence
Oriti, 2021 [57]	AR glasses (HoloLens)	IV: none DV: involvement in the history; social presence
Lee, 2021 [35]	AR glasses (HoloLens)	IV: none DV: co-presence
Georgiou, 2021 [58]	Tablet	IV: condition (strongly-coupled vs. loose) DV: immersion
Jin, 2021 [59]	AR glasses (HoloLens)	IV: user interface-UI (natural UI vs. graphical UI) DV: presence
Kosa, 2022 [60]	Smartphone	IV: none DV: presence

**Table 5:** List of evaluations conducted across the analysed records.

Reference	Evaluation Tools	Sample Size	Reference for Evaluation
McCall, 2011 [11]	Questionnaires; interviews; observation	24	Modified MEC spatial presence questionnaire [64] by adding some questions from a social presence questionnaire [65]
Putten, 2012 [45]	Questionnaires; interviews; observation	44	Modified MEC spatial presence questionnaire [64] by adding some questions from a social presence questionnaire [65]
Furió, 2013 [50]	Questionnaires	79	
Datcu, 2016 [41]	Questionnaires	18	Adapted AR presence questionnaire [66]
Koh, 2017 [13]	Questionnaires	459	
Oh, 2018 [51]	Questionnaires; interviews	20	Custom
Raptis, 2018 [52]	Questionnaires; interviews	73	Immersive Experience Questionnaire (IEQ) [24]
Sekhavat, 2018 [27]	Questionnaires	30	Immersive Experience Questionnaire (IEQ) [24]
Bueno, 2020 [15]	Questionnaires	1183	Custom
Estudante, 2020 [53]	Questionnaires	70	
Krzywinska, 2020 [26]	(Not enough data)	39	
Lin, 2020 [54]	(Not enough data)	52	
Rossano, 2020 [55]	Questionnaires	33	User Engagement Scale (UES) short form [67]
Seaborn, 2020 [56]	Questionnaires	37	Game Engagement Questionnaire (GEQ) [28]; and Player Experience of Need Satisfaction (PENS) [68]
Shin, 2021 [36]	Questionnaires	281	Custom [69]
Oriti, 2021 [57]	Questionnaires	20	
Lee, 2021 [35]	Questionnaires	34	Game Experience Questionnaire [70]
Georgiou, 2021 [58]	Questionnaires	45	Augmented Reality Immersion (ARI) Questionnaire [46]
Jin, 2021 [59]	Questionnaires	58	Presence Questionnaire [31]; Narrative Engagement Scale [71]
Kosa, 2022 [60]	Questionnaires	49	Adapted Player Experience of Need Satisfaction (PENS) [68]

## Data Availability

Not applicable.

## Abbreviations

The following abbreviations are used in this manuscript:

AR	Augmented Reality
ARI	Augmented Reality Immersion
DV	Dependent Variable
GEQ	Game Engagement Questionnaire
IEQ	Immersive Experience Questionnaire
IV	Independent Variable
PENS	Player Experience of Need Satisfaction
RPG	Role-Playing Game
UES	User Engagement Scale
VR	Virtual Reality
UI	User Interface
